# Genome-Wide Identification and Expression Analysis of Calcium-dependent Protein Kinase in Tomato

**DOI:** 10.3389/fpls.2016.00469

**Published:** 2016-04-08

**Authors:** Zhangjian Hu, Xiangzhang Lv, Xiaojian Xia, Jie Zhou, Kai Shi, Jingquan Yu, Yanhong Zhou

**Affiliations:** ^1^Department of Horticulture, Zhejiang UniversityHangzhou, China; ^2^Zhejiang Provincial Key Laboratory of Horticultural Plant Integrative BiologyHangzhou, China

**Keywords:** CDPK, environmental stimuli, gene family, phytohormone, *Solanum lycopersicum*, transcript

## Abstract

Calcium-dependent protein kinases (CDPKs) play critical roles in regulating growth, development and stress response in plants. Information about CDPKs in tomato, however, remains obscure although it is one of the most important model crops in the world. In this study, we performed a bioinformatics analysis of the entire tomato genome and identified 29 CDPK genes. These CDPK genes are found to be located in 12 chromosomes, and could be divided into four groups. Analysis of the gene structure and splicing site reflected high structure conservation within different CDPK gene groups both in the exon-intron pattern and mRNA splicing. Transcripts of most CDPK genes varied with plant organs and developmental stages and their transcripts could be differentially induced by abscisic acid (ABA), brassinosteroids (BRs), methyl jasmonate (MeJA), and salicylic acid (SA), as well as after exposure to heat, cold, and drought, respectively. To our knowledge, this is the first report about the genome-wide analysis of the CDPK gene family in tomato, and the findings obtained offer a clue to the elaborated regulatory role of CDPKs in plant growth, development and stress response in tomato.

## Introduction

Plants have evolved different strategies to acclimatize themselves to various challenging environments, including biotic and abiotic stresses. Hereinto calcium (Ca^2+^) participates in miscellaneous signal transduction pathways as the second messenger (Boudsocq et al., 2012). Calcium sensors or calcium-binding proteins can recognize transient calcium concentration variations, and in turn alter transcript of downstream genes, proteins phosphorylation or enzyme activity (Sanders et al., 1999; Harmon et al., 2000). To date, three classes of calcium sensors have been characterized in plants: calmodulin (CaM) and CaM-like protein (CaML), calcineurin B-like protein (CBL), and calcium-dependent protein kinases (CDPKs). Among these calcium sensors, only CDPKs, depending on their special structure, can directly sense, respond, and translate Ca^2+^ signals into downstream protein phosphorylation without protein partner conformational change (Cheng et al., 2002; Ludwig et al., 2004; Harper and Harmon, 2005; Poovaiah et al., 2013).

The basal architecture of the CDPK family shows highly conserved in land plant, even in bryophyte and pteridophyte (Hamel et al., 2014). They display a conserved molecular structure with four characterized domains, *N*-terminal domain, Ser/Thr kinase domain, autoinhibitory junction domain, and calmodulin-like domain (Hrabak et al., 2003). Different species, even different CDPK variants in the same species, have distinguished length of *N*-terminal domain (Ito et al., 2010), which is associated to protein localization and *in vivo* phosphorylation (Schulz et al., 2013). The Ser/Thr kinase domain is the catalytic domain with ATP binding site, which plays a central role for the function of CDPK. The intracellular calcium concentrations could significantly modulate CDPK activity (Harper et al., 2004; Boudsocq et al., 2012), sub-cellular localization (Raichaudhuri et al., 2006), and the interactions with other proteins (Ishida et al., 2008; Zou et al., 2010). The autoinhibitory junction domain found in CDPK at CDPK-SnRK superfamily is implied as an autoinhibitor to maintain CDPK inactive or to be activated by stimulation through typical cytosolic calcium signals, which leads CDPK activation as a relieving autoinhibition mechanism (Yang and Poovaiah, 2003; Klimecka and Muszynska, 2007). The C-terminal calmodulin-like domain of CDPKs holds up to four elongation factor (EF) hands, a 29 aa helix-loop-helix structure, which owns 13 conserved residues to sense calcium signals and bind to the free calcium ion (Harmon, 2003). Taken CDPK autoinhibition mechanism as a consideration, a truncated CDPK form, without the autoinhibitory junction domain and calmodulin-like domain exhibits constitutive activity in the absence of calcium (Harper et al., 1994).

Except for calcium signal, other regulatory components, like 14-3-3 proteins (Camoni et al., 1998; Lachaud et al., 2013), phospholipids (Dixit and Jayabaskaran, 2012), and sometimes reversible phosphorylation and/or autophosphorylation (Cheng et al., 2002; Chehab et al., 2004; Hegeman et al., 2006) can also regulate CDPKs activity. On the other hand, CDPKs could interact with and modified downstream targets involved in abiotic tolerance, anti-pathogens response, plant immunity, hormonal signal transduction, plant growth and development, pollen germination and seed development (Boudsocq and Sheen, 2013; Schulz et al., 2013). For example, *At*CPK10 is likely to regulate the stomata movement in response to drought through interacting with HSP1 in *Arabidopsis* (Zou et al., 2010). In *Arabidopsis* guard cells, *At*CPK6/21/23 interacted with *At*SLAC1 and participated in abscisic acid (ABA)-induced stomatal closure (Geiger et al., 2010; Brandt et al., 2012). In tomato, *Le*CDPK2 could modulate the wounding signaling by phosphorylating *Le*ACS2 for controlling the ethylene production (Kamiyoshihara et al., 2010). Recent research has revealed reduplicate roles of CDPKs in nucleotide-binding domain leucine-rich repeat (NLR) immune signaling. *At*CPK4/11 could directly phosphorylate *At*WRKY8/28/48 in response to pathogen infection while *At*CPK4/11 could phosphorylate *At*RBOHD and *At*RBOHF in the generation of reactive oxygen species (ROS; Gao et al., 2013). Though the big family of CDPK genes showed high functional redundancy and little morphological diversity in single mutants, the anatomical observation of *Atcpk28* mutant proved *At*CPK28 is involved in plant stem and petiole elongation, and vascular development (Matschi et al., 2013). Recently, *At*CPK28 was found to be involved in the regulation of plant immunity through phosphorylating BIK1 and regulating its turnover (Monaghan et al., 2014). Besides, *At*CPK17/34 were found to be the pivotal regulators in pollen tube tip growth (Myers et al., 2009). *At*CPK12 boosted seeding germination as a negative factor in ABA-signaling, while overexpression of *OsCDPK2* in rice plants resulted in disruption of seed development (Morello et al., 2000; Zhao et al., 2011).

As the plant special protein kinases, CDPKs consist of multigene family. There are 34 CDPK genes in *Arabidopsis* (*Arabidopsis thaliana*; Cheng et al., 2002; Hrabak et al., 2003), 29 CDPK genes in rice (*Oryza sativa*; Asano et al., 2005), 20 CDPK genes in wheat (*Triticum aestivum*; Li et al., 2008), 40 CDPK genes in maize (*Zea mays*; Kong et al., 2013), 30 CDPK genes in poplar (*Populus trichocarpa*; Zuo et al., 2013), 17 CDPK genes in grapevine (*Vitis vinifera*; Chen et al., 2013), 41 CDPK genes in diploid cotton (*Gossypium raimondii*; Liu et al., 2014), respectively. Based on long time evolution view, CDPK family exhibited highly conserved architecture from bryophytes to higher species (Hamel et al., 2014). Gene duplication is the main reason for the expansion of the CDPK family. Notably evidence shown in *Arabidopsis*, *CPK21/22/23/27/31* are tandem duplicated in Chromosome IV (Cheng et al., 2002). Besides, some duplication specifically occurs in certain species, like *AtCPK18* and *OsCPK4* verse *CPK39/40* in maize (Kong et al., 2013). All land plant CDPK gene family members could be clustered into four groups based on their phylogenetic relationship. Among them, Group IV has smallest amount of CDPK gene members (Cheng et al., 2002; Hrabak et al., 2003; Asano et al., 2005; Li et al., 2008; Chen et al., 2013; Kong et al., 2013; Zuo et al., 2013; Liu et al., 2014). Meanwhile, Group IV is more close to ancient algae CDPK genes as compared to Group I, II, and III (Hamel et al., 2014).

Until now, no systematic genome-wide identification and expression analysis of CDPK family genes have been carried out in vegetable crops and so far, only four CDPK genes, *LeCDPK1* (same to *SlCDPK18* in this article), *LeCPK1* (same to *SlCDPK16* in this article), *LeCDPK2* (same to *SlCDPK4* in this article) and *LeCPK2* (same to *SlCDPK29* in this article), have been characterized in tomato. These CDPK genes were found to be responsive to wounding, heat stress, and hormones, respectively (Chico et al., 2002; Rutschmann et al., 2002; Chang et al., 2009; Kamiyoshihara et al., 2010). In this regard, the completion of tomato genome sequencing (Sato et al., 2012) will greatly aid us to explore their role in plant growth, development and stress response in tomato.

In this study, we performed bioinformatics analysis of the whole tomato genome and identified 29 CDPK genes. These tomato CDPKs were grouped based on their phylogenetic relationships and were anchored to specific chromosomes. Furthermore, we analyzed the transcript of 28 CDPK genes in various organs at different developmental stages and their response to plant hormones, like ABA, brassinosteroids (BRs), methyl jasmonate (Me-JA), and salicylic acid (SA) as well as to several typical abiotic stresses heat, cold, and drought.

## Materials and Methods

### Identification of Tomato CDPK Genes

To comprehensively annotate CDPK genes in tomato, all reported 34 *Arabidopsis* and 29 rice CDPK protein sequences were retrieved from The *Arabidopsis* Information Resource^1^ and Rice Genome Annotation Database^2^. The 63 sequences from *Arabidopsis* and rice were blasted against the Sol Genomics Network^3^ and Tomato genome sequencing project^4^ databases to obtain putative CDPK sequences in tomato. The putative CDPK candidate genes were analyzed with ScanProsite^5^, Conserved Domain Database^6^, and Pfam^7^ for conserved CDPK domains and structure.

### Chromosomal Location, Gene Structure, Splicing Sites and Phylogenetic Tree of CDPKs

Calcium-dependent protein kinases from *Arabidopsis*, rice, and tomato were aligned by Clustal X 2.01 program in default settings (Larkin et al., 2007). Phylogenetic tree was built under MEGA5.03 program using the maximum-like hood method (Tamura et al., 2011). Chromosomal location of CDPKs was determined by the information achieved from Tomato genome sequencing project. Based on the tomato genome information, exon/intron organizations of 29 individual CDPK genes were depicted with the help of Gene Structure Display Server^8^. Splicing sites were depicted into CDPK protein structure provided by ScanProsite and Pfam according to exon/intron end sites.

### Forecast of the Ratios of *K*_a_/*K*_s_ in Tomato CDPK Paralogous Gene Pair

A total of eight pairs of CDPK paralogous genes were selected based on the query coverage and identity values over 80%. Only the tightly linked genes were qualified for one duplication event. The paralogous genes were aligned based on coding sequence with the Clustal W program in MEGA 5.03. The means of DnaSP v5.0 software, non-synonymous substitutions per non-synonymous site (*K*_a_) to the number of synonymous substitutions per synonymous site (*K*_s_) were calculated accordingly (Librado and Rozas, 2009).

### Plant Materials and Growth Conditions

Tomato (*Solanum lycopersicum* L. cv. MicroTom) plants were cultured in growth chambers, which were maintained at a 16 h light (200 μmol m^-2^ s^-1^) at 25°C and an 8 h dark at 19°C, respectively. For the analysis of the transcripts of CDPK genes, 3-month-old tomato plants were used. Roots, stems, leaves, flowers, and fruits at different developmental stages were collected for RNA extraction.

To analyze the response of CDPK genes to different phytohormones at the transcriptional level, leaves of 8-week-old tomato plants were sprayed with ABA at 100 μM, 24-epibrassinolite (EBR) at 200 nM, Me-JA at 100 μM, SA at 2 mM, and water, respectively. Leaves samples were collected for RNA extraction at 0, 0.5, 1, 3, and 9 h, respectively. Leaves with water treatment at 0 h were used as the control.

To analyze the response of CDPK genes to different abiotic stresses at the transcriptional level, 8-week-old tomato plants were exposed to 45°C (high temperature), 4°C (low temperature), or drought by water withdrawing, respectively. Plants grown at 25°C with well irrigated were used as control. For the high temperature and low temperature treatments, leaves were sampled at 1, 3, and 9 h, respectively, after the treatment. For the drought treatment, leaf samples were taken at 1, 3, and 6 days, respectively.

### Total RNA Extraction and Gene Expression by Real-Time Quantitative RCR

Total RNA was isolated from tomato leaves using TRIZOL reagent (Sangon, China) according to the instructions supplied by the manufacturer. After extraction, total RNA was dissolved in diethyl pyrocarbonate-treated water. The cDNA template for real time RT-PCR was synthesized using a ReverTra Ace qPCR RT Kit (Toyobo, Osaka, Japan) from 2 μg total RNA.

For quantitative RT-PCR analysis, we amplified PCR products in triplicate using iQ SYBR Green SuperMix (Bio-Rad, Hercules, CA, USA) in 25 μL qRT-PCR reactions. PCR was performed using the iCycleriQ 96-well real-time PCR Detection System (Bio-Rad) and cycling conditions consisted of denaturation at 95°C for 3 min, followed by 40 cycles of denaturation at 95°C for 30 s, annealing at 58°C for 30 s and extension at 72°C for 30 s. The tomato actin gene was used as an internal control. Gene-specific primers were designed and used for amplification as described in Supplementary Table S1. Relative gene expression was calculated as described by Livak and Schmittgen (2001).

## Results And Discussion

### Genome-Wide Identification of CDPK Gene Family in Tomato

BLAST searches of CDPKs in tomato genome (Tomato Genome Sequencing Project, 2012) were performed against the *Arabidopsis*, rice and maize CDPK gene sequences. This allowed us to identify 29 putative CDPK genes, designated as *SlCDPK1-SlCDPK29* following the proposed nomenclature for CDPK genes (**Table 1**). All the 29 CDPKs had conserved CDPK domains, a variable *N*-terminal domain, a Ser/Thr kinase domain, an autoinhibitory junction domain, and a calmodulin-like domain. A variable *N*-terminal domain was present in all of 29 *Sl*CDPK genes with their open reading frame (ORF) length ranging from 1290 (*SlCDPK15*) to 1749 (*SlCDPK2*), which encoded polypeptides in the range of 429 to 598 aa, with predicted protein molecular mass from 48.06 to 67.55 kDa, respectively (**Table 1**). Interestingly, the isoelectric point of all *Sl*CDPKs tended to be acidic with the exception of 3-EF-hands deficient *SlCDPK15* and *SlCDPK28/29*, which showed isoelectric point alkalinity. Meanwhile, 23 of the 29 *Sl*CDPKs contained predicted myristoylation site and 15 *Sl*CDPKs had putative palmitoylation site (**Table 1**). Although the *N*-terminal sequences varied with CDPKs, *SlCDPK23-SlCDPK29* all had the myristoylation site, which was similar to those in *Arabidopsis* and maize (Boudsocq and Sheen, 2013; Kong et al., 2013). As *N*-myristoylation promotes protein-membrane and protein–protein interaction, while as a second lipid modification, *N*-palmitoylation can stabilize the interaction between protein and membrane (Martin and Busconi, 2000), these CDPKs may function in many physiological processes by membrane association in plants.

**Table 1:** Characteristics of calcium-dependent protein kinases (CDPKs) in tomato.

Name	Locus name	Group	Deduced polypeptide			Position	*N*-acylation prediction	No. of EF-hands
			ORF length (bp; aa)	(PI)	MW(KD)			
CDPK1	Solyc01g006730.2.1	I	1563/520	5.74	64.62	1332580-1328459	–	4
CDPK2	Solyc01g006840.2.1	I	1797/598	5.34	67.55	1414084-1410577	*N*-Myr	4
CDPK3	Solyc01g112250.2.1	I	1605/534	5.59	59.92	90084923-90090825	*N*-Myr	4
CDPK4	Solyc04g009800.2.1	I	1746/581	5.54	64.60	3114299-3108503	*N*-Myr	4
CDPK5	Solyc04g049160.2.1	I	1527/508	5.11	57.23	39080746-39092319	–	4
CDPK6	Solyc05g056570.2.1	I	1512/503	5.02	56.43	64966125-64971506	–	4
CDPK7	Solyc06g065380.2.1	I	1524/507	5.69	57.14	37191712-37195833	–	4
CDPK8	Solyc10g074570.1.1	I	1674/557	5.55	62.25	57454225-57458658	*N*-Myr	4
CDPK9	Solyc10g076900.1.1	I	1506/501	5.31	55.62	59195105-59198922	*N*-Myr	4
CDPK10	Solyc10g081640.1.1	I	1740/579	5.01	63.52	62001043-62005206	*N*-Myr	4
CDPK11	Solyc10g081740.1.1	I	1500/499	5.08	55.76	62086600-62082038	–	4
CDPK12	Solyc11g006370.1.1	I	1737/578	5.45	64.68	1080169-1085490	*N*-Myr	4
CDPK13	Solyc11g018610.1.1	I	1518/505	5.38	56.89	8769972-8773835	–	4
CDPK14	Solyc01g008740.1.1	II	1626/541	5.49	61.10	2776811-2782126	*N*-Myr-Palm	4
CDPK15	Solyc02g032820.2.1	II	1290/429	8.97	48.60	20070295-20078341	*N*-Myr-Palm	1
CDPK16	Solyc03g031670.2.1	II	1662/553	6.32	62.99	8406044-8397378	*N*-Myr-Palm	4
CDPK17	Solyc04g081910.2.1	II	1566/521	5.29	58.92	63365446-63370378	*N*-Myr-Palm	4
CDPK18	Solyc07g064610.2.1	II	1566/521	6.59	57.82	63903803-63909109	*N*-Myr-Palm	4
CDPK19	Solyc08g008170.2.1	II	1551/516	5.75	57.70	2642478-2637882	*N*-Myr-Palm	4
CDPK20	Solyc11g064900.1.1	II	1590/529	5.74	59.53	47276122-47269846	*N*-Myr-Palm	4
CDPK21	Solyc12g099790.1.1	II	1608/535	5.39	59.63	65130565-65133955	*N*-Myr-Palm	4
CDPK22	Solyc01g008440.2.1	II	1602/533	6.55	59.99	2523675-2529463	*N*-Myr-Palm	4
CDPK23	Solyc03g113390.2.1	III	1617/538	6.43	60.94	57593080-57598567	*N*-Myr-Palm	4
CDPK24	Solyc06g073350.2.1	III	1611/536	5.70	61.06	41593571-41589654	*N*-Myr-Palm	4
CDPK25	Solyc09g005550.2.1	III	1590/529	6.04	59.62	377428-369215	*N*-Myr-Palm	3
CDPK26	Solyc10g079130.1.1	III	1578/525	5.92	59.63	60068542-60064941	*N*-Myr-Palm	3
CDPK27	Solyc11g065660.1.1	III	1602/533	6.03	59.66	48279107-48274194	*N*-Myr-Palm	3
CDPK28	Solyc02g083850.2.1	IV	1713/570	9.31	64.20	41695031-41690032	*N*-Myr	4
CDPK29	Solyc03g033540.2.1	IV	1698/565	9.03	63.88	9363698-9356208	*N*-Myr-Palm	4

At the *C*-terminal CaM-like domain, a typical CDPK embraces up to 4 EF hand motifs. A detailed analysis revealed that *SlCDPK22/25/26/27* had three EF hand motifs, and others had common four EF hand motifs. However, *SlCDPK15* only had one EF hand like *AtCPK25*, *OsCPK6*, *ZmCPK7*, and *PtCDPK15*. EF hands of CDPKs act as a calcium sensor in calcium-binding affinities (Harper et al., 2004; Harper and Harmon, 2005; Liese and Romeis, 2013). In agreement with this, *AtCPK25* was calcium independent owing to lacking of the functional EF hands (Boudsocq et al., 2012). Therefore, these CDPKs like *SlCDPK15* might be insensitive to the changes in cellular calcium in plants.

### CDPK Gene Chromosomal Distribution and Phylogenetic Analysis in Tomato

**FIGURE 1:**
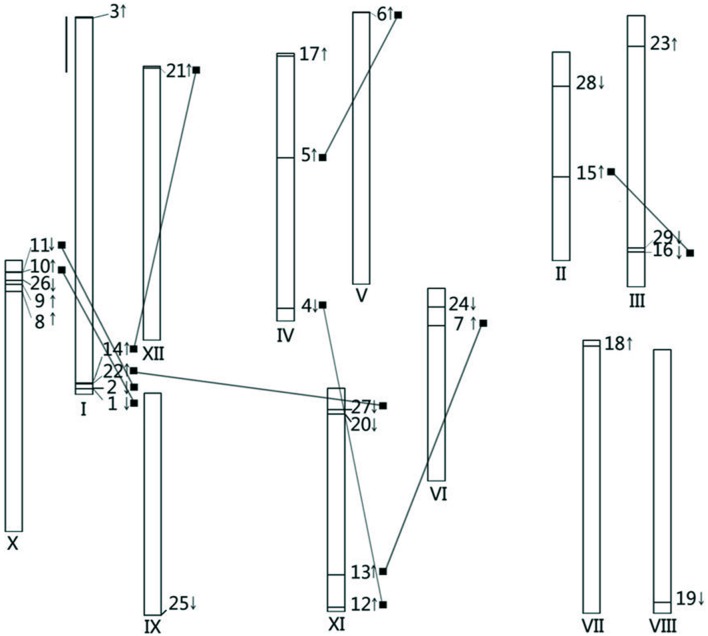
**Chromosomal distributions of calcium-dependent protein kinases (CDPK) genes in the tomato genome.** The roman numerals under each chromosome represent the nomber of the chromosomes. The arrows accompanied with each CDPK gene stand for the gene encoding direction. Each of paralogous gene pair is linked by the black line. The black bar on the left up concern means the length of 10 Mb.

The 34 and 29 CDPK genes in *Arabidopsis* and rice, respectively, were distributed among all 5 and 12 chromosomes of their respective genomes (Cheng et al., 2002; Kong et al., 2013; Zuo et al., 2013; Liu et al., 2014). *In silico* chromosomal localization of CDPKs revealed that 29 *Sl*CDPK genes were anchored in all of the 12 chromosomes, which implied multigene family functions in tomato plants (**Figure 1**). Chromosome I and chromosome X of tomato both had up to 5 *Sl*CDPK genes but only one unique *Sl*CDPK gene was distributed in each of chromosome V, chromosome VII, chromosome VIII, chromosome IX, and chromosome XII, respectively. Besides, *SlCDPK8/9/26/10/11* and *SlCDPK1/2/22/14* were separately clustered on the edge of chromosome I and chromosome X. Interestingly, eight paralogous gene pairs were dispersedly distributed in two different chromosomes. However, no paralogous gene pairs were found in chromosome VII/VIII/IX. All these results suggested that CDPK genes are widely distributed in tomato genomes.

**FIGURE 2:**
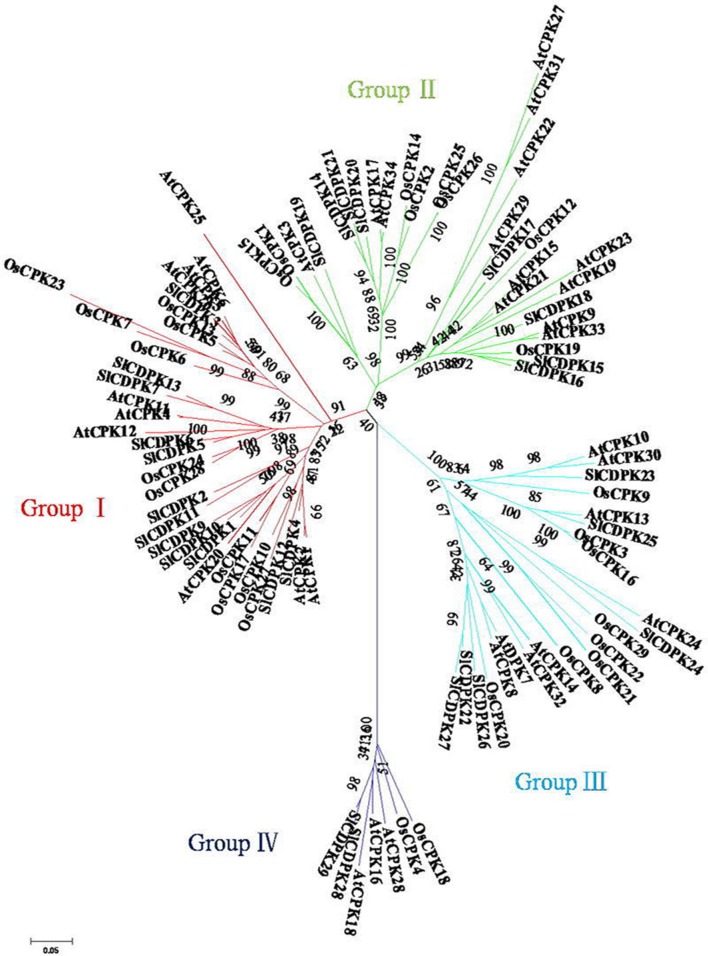
**Phylogeny and distribution of CDPK proteins from *Arabidopsis*, rice, and tomato.** Methods follow neighbor-joining with 1000 bootstrap in MEGA 5.0 according to full length amino acid sequences of 29 tomato, 34 *Arabidopsis*, and 29 rice CDPKs.

To dissect the evolutionary relationships of CDPK family members, the dicot model plant *Arabidopsis*, the monocot model plant rice, and tomato CDPK full-length amino acid sequences were used to construct an unrooted tree (**Figure 2**). The phylogenetic analysis clearly showed all 29 *Sl*CDPKs could be divided into four groups (Group I–Group IV), like that in *Arabidopsis* and rice CDPKs. Up to 13 tomato CDPKs together with 11 rice CDPKs and 10 *Arabidopsis* CDPKs belonged to Group I, which made it to be the largest group in tomato CDPK family. Group II contained 7 CDPK members from tomato, 8 from rice, and 13 from *Arabidopsis*. Meanwhile, 6 *Sl*CDPKs, 8 *Os*CDPKs, and 8 *At*CDPKs were within the family of Group III, respectively. Finally, Group IV was the smallest family, and it only contained 2 tomato CDPKs (*SlCDPK28/29*), 3 *Arabidopsis* CDPKs (*AtCDPK16, AtCDPK18*, and *AtCDPK28*), and 2 rice CDPKs (*OsCDPK4* and *OsCDPK18*).

Based on the phylogenetic map, a total of eight pairs *Sl*CDPK genes were presented as paralogous gene pairs, and all of these paralogous gene pairs showed higher than 80% similarity in nucleotide sequence, indicating an overlap of these CDPKs in function (**Table 2**). As showed in CDPK chromosomal distribution (**Figure 1**), all of these eight paralogous gene pair belonged to segmental duplications rather than tandem duplications, suggesting that segmental duplication caused *Sl*CDPK gene family expansion. Furthermore, by calculating the ratio of *K*_a_/*K*_s_, an indicator for the selection history of these paralogous gene pairs (Yang et al., 2006), we found that the *K*_a_/*K*_s_ of eight paralogous gene pairs was lower than 0.2, suggesting that these gene pairs had strong purifying selection stress, and made the function of these gene pairs trend to be relative similar.

**Table 2:** *K*_a_/*K*_s_ ratios reflected in CDPK paralogous pairs.

Paralogous pairs	Identities (%)	*K*_a_	*K*_s_	*K*_a_/*K*_s_	Purifying selection
*Sl*CDPK2/11	83	0.089	0.766	0.116	Yes
*Sl*CDPK4/12	88	0.069	0.501	0.137	Yes
*Sl*CDPK5/6	89	0.061	0.532	0.115	Yes
*Sl*CDPK7/13	87	0.070	0.646	0.108	Yes
*Sl*CDPK15/16	85	0.155	0.830	0.187	Yes
*Sl*CDPK22/27	87	0.061	0.591	0.102	Yes
*Sl*CDPK1/10	87	0.113	0.685	0.165	Yes
*Sl*CDPK14/21	84	0.103	0.939	0.109	Yes

### CDPK Genes Structure and Splicing Sites in Tomato

**FIGURE 3:**
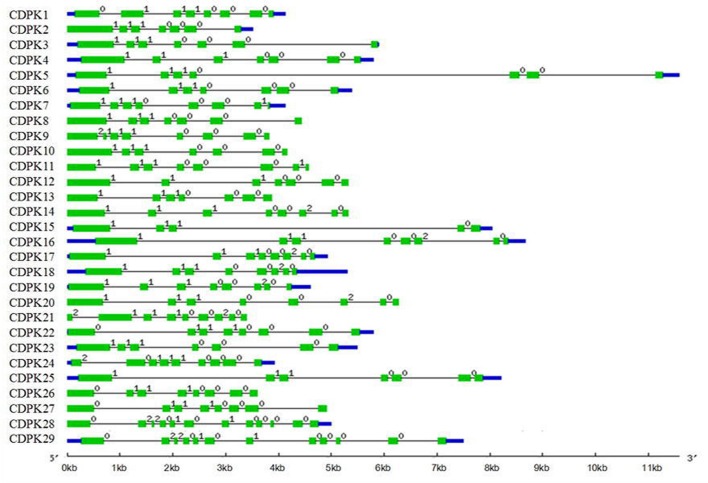
**Gene structure in tomato CDPK gene family.** Blue bars stand for untranslated regions (UTR). One green bars stand for an exon and one black string stand for an intron. The number (0, 1, and 2) above the black strings stand for intron phase.

**FIGURE 4:**
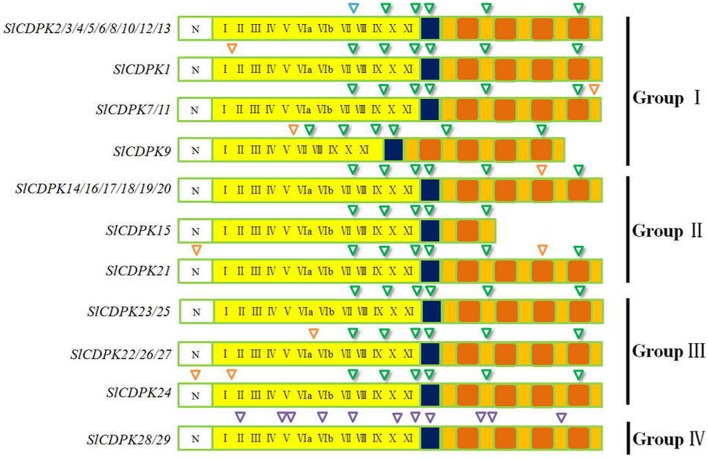
**Splicing sites of CDPK genes in tomato.** Green inverted triangles represent the basic splicing sites in CDPK genes. Orange inverted triangles represent the special splicing sites in CDPK genes. Purples inverted triangles represent the unique splicing sites in Group IV CDPK genes.

As the most abundant serine/threonine kinases in plants, CDPK gene family presented high hereditary conservation (Sheen, 1996). The exon-intron structure and splicing site mapping are essential approaches for discovering family gene structure diversity and evolutionary divergence. Based on coding DNA sequence of *Sl*CDPK genes and completed tomato genome sequence, maps of tomato CDPK exon-intron structure (**Figure 3**) and splicing sites were integratively depicted (**Figure 4**). In general, the total intron numbers varied from 4 (*SlCDPK15*) to 11 (*SlCDPK28*/*29*) in 29 *Sl*CDPK genes, as observed in rice, maize, and *Arabidopsis*. Majority of *Sl*CDPK genes from Group I to Group III had six introns in their unsplicing sequences, and even showed the same intron phase. Other extra introns were inserted mainly in four different ways. Firstly, an extra intron was inserted in *N*-terminal domain of *SlCDPK21/24*. Secondly, different subarea of kinase domain in*SlCDPK1/24*, *SlCDPK9*, and *SlCDPK22/26/27* exist another intron. Thirdly, almost all *Sl*CDPK genes from Group II had the extra intron in third EF hand motif expect for *SlCDPK15*. Finally, *SlCDPK7/11* showed a unique intron in other part of CaM-like domain. In spite of the diverse length of introns and different numbers of exons, almost all the *Sl*CDPK genes from Group I to Group III owned six basic exons, which utilized the identical splicing sites at almost the same point, except for structure-deficient *SlCDPK15* which lacked three EF-hands in CaM-like domain. Like *SlCDPK15*, *SlCDPK9* was another particular member because of its incomplete kinase domain. Meanwhile, *SlCDPK28/29* had totally different gene splicing structures (**Figure 4**), and the gene structure of Group IV has been reported more similar to CDPK-related kinases (CRK; Zuo et al., 2013), suggesting a high conservation of this family through evolution.

### Expression of Tomato CDPK Genes in Different Plant Tissues

**FIGURE 5:**
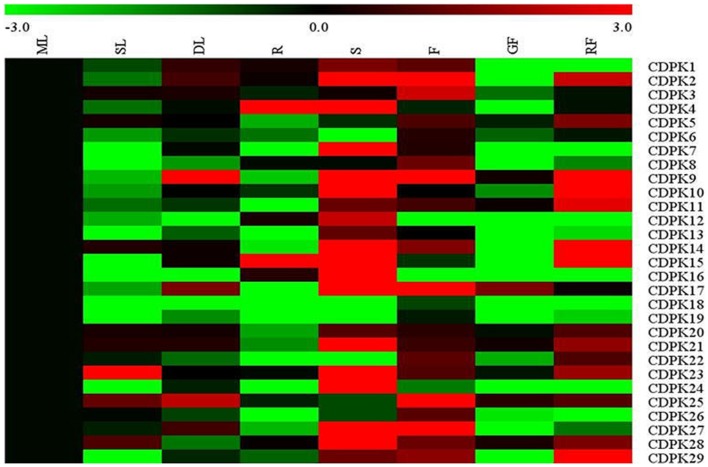
**Expression profiles of CDPK genes across different tissues in tomato.** ML, mature leave; SL, senescence leave; DL, developing leave; R, root; S, stem; F, flower; GF, green fruit; RF, red fruit. The color scale stands for the relative signal intensity values.

To characterize CDPK transcript patterns in different organs of tomato plants, we synthesized 28 corresponding primer pairs for 29 tomato CDPK genes except for *CDPK20*, which was not accurately found (**Figure 5**). As found in **Figure 5**, the transcript of most CDPK genes was organ-dependent. In agreement with those observed in poplar and maize (Kong et al., 2013; Zuo et al., 2013), transcript for most CDPK genes was highest in stems, followed by those in red fruits and flowers, respectively. In comparison, senescence leaves, roots and green fruits showed decreased transcripts of most CDPKs as compared to those in mature leaves. It is interesting to note that transcripts of most CDPK genes were down-regulated in senescence leaves as compared to mature leaves whilst transcripts of most these genes were up-regulated in the red fruits. As observed in earlier study, we also found high transcriptional level of *SlCDPK29* in flower (Chang et al., 2009).

### CDPK Transcript in Response to Exogenous Phytohormones

**FIGURE 6:**
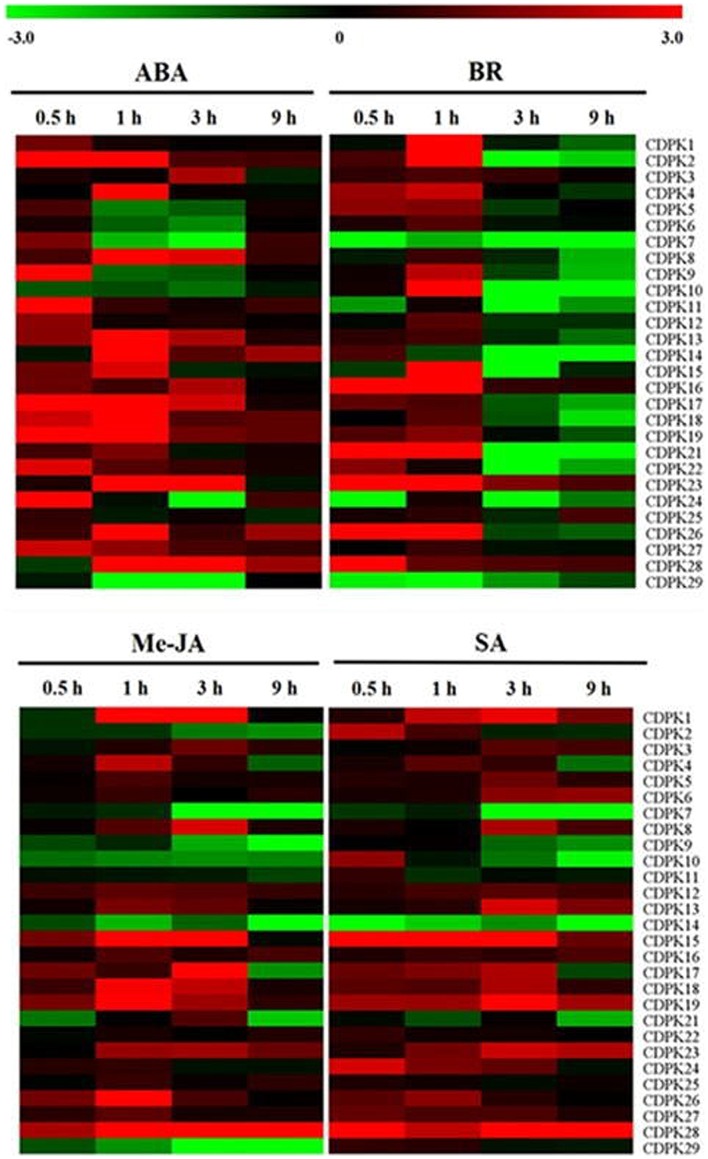
**Expression analysis of 28 CDPK genes in leaves of tomato in response to phytohormones.** Leaves were exposed to ABA at 100 μM, EBR at 200 nM, MeJA at100 μM and SA at 2 mM and sampled at various times for the quantitative real-time RT-PCR analysis. The color scale stands for the relative signal intensity values.

Increasing studies have shown the crosstalk between CDPK and phytohormones in plant defense and development processes (Yang and Komatsu, 2000; Ludwig et al., 2005; Zhao et al., 2011; Yang et al., 2012; Ding et al., 2013). Here we found that the transcript of these *Sl*CDPK genes responded differentially to exogenous phytohormones (**Figure 6**). In response to exogenous ABA, transcripts of an overwhelming majority of *Sl*CDPK genes were up-regulated within 0.5 h and lasted until to 3 h, eventually dropped to the control level in 9 h. It seems likely that transcripts of *Sl*CDPK genes from Group II were more significantly up-regulated in response to ABA which is in agreement with the changes in *AtCPK3/23* in Group II of *Arabidopsis* (Mori et al., 2006; Geiger et al., 2010). However, transcript of *SlCDPK7*, which is a homolog gene *ZmCPK14* functioning as an ABA negative regulator (Kong et al., 2013) in maize, was down-regulated by ABA, at 1 and 3 h. In addition, *SlCDPK5/6* kept at a low transcript level after ABA treatment, and their homologous *AtCPK12* is known to negatively modulate ABA signaling in seeding germination and growth (Zhao et al., 2011). In *Arabidopsis*, it has been reported CDPKs were involved in ABA signal pathways by two ABA-associated factors. One of them is ABA-responsive element binding factors (ABFs), such like ABF1 and ABF4, which could be phosphorylated by certain CDPKs, as CPK4 and CPK11, positively participated in CDPK-induced ABA pathways (Zhu et al., 2007). The other showed that suppressed PP2C-type phosphatases like ABI1 and ABI2 protein could activate certain CDPKs-induced stomatal closure after ABA treatment (Geiger et al., 2010). However, the role of these *Sl*CDPKs in ABA signaling remains to be studied in tomato.

Brassinosteroids (BRs) are noted for having a broad-spectrum of physiological functions; meanwhile crosstalk and interactions of BRs and other hormones seemed to be pervasive in stress response (Choudhary et al., 2012). In our study, lots of *Sl*CDPK genes were stimulated at transcript levels only within 1 h, but suppressed then after foliar application of EBR and this is especially apparent in *SlCDPK2*, *SlCDPK10*, *SlCDPK15*, and *SlCDPK21*. However, transcripts of *SlCDPK6* and *SlCDPK27* showed null response to exogenous EBR whist transcript of *SlCDPK7* was down-regulated by EBR throughout the times. Until now, little is known about the CDPK in BR signaling although a few studies demonstrated that some CDPKs may play a role as downstream components of the BRs receptor in rice (Yang and Komatsu, 2000; Sharma et al., 2001).

Jasmonate (JA)- and SA-mediated defense pathways were two important defense mechanisms for plant against microorganisms and herbivores, including bacteria, oomycetes, fungi, and insects (Dong, 1998). There are evidences that CDPKs are involved in these responses (Boudsocq and Sheen, 2013). In our study, transcripts of most CDPK genes showed similar response to exogenous SA and JA and both SA and JA induced transcripts of a majority of *Sl*CDPK genes (**Figure 6**). In rice, overexpression of *OsCPK10* or *OsCPK20* induced the transcript of SA- and JA-related genes and resistance against *Pseudomonas syringae* pv. *tomato* and *Magnaporthe grisea* (Fu et al., 2013, 2014). Recently, it has been reported *Arabidopsis cpk28* mutant regulated development by inducing JA (Matschi et al., 2015). Nevertheless, *Sl*CDPK genes of Group I seemed to be less sensitive to SA and JA as indicated by less induction in their transcript at 1 h as compared to those in other groups. Interestingly, transcripts of *SlCDPK7* and *SlCDPK14* were downregulated by both SA and JA while transcript of *SlCDPK29* was downregulated by SA but not altered by JA. Similarly, transcript of *SlCDPK2* was downregulated by SA but induced as soon as 1 h after SA application. The different response of the transcripts of these *Sl*CDPK genes to JA and SA suggested their potential role in the resistance against biotic stress.

### Expression Profiles of Tomato CDPK Genes under Abiotic Stress

**FIGURE 7:**
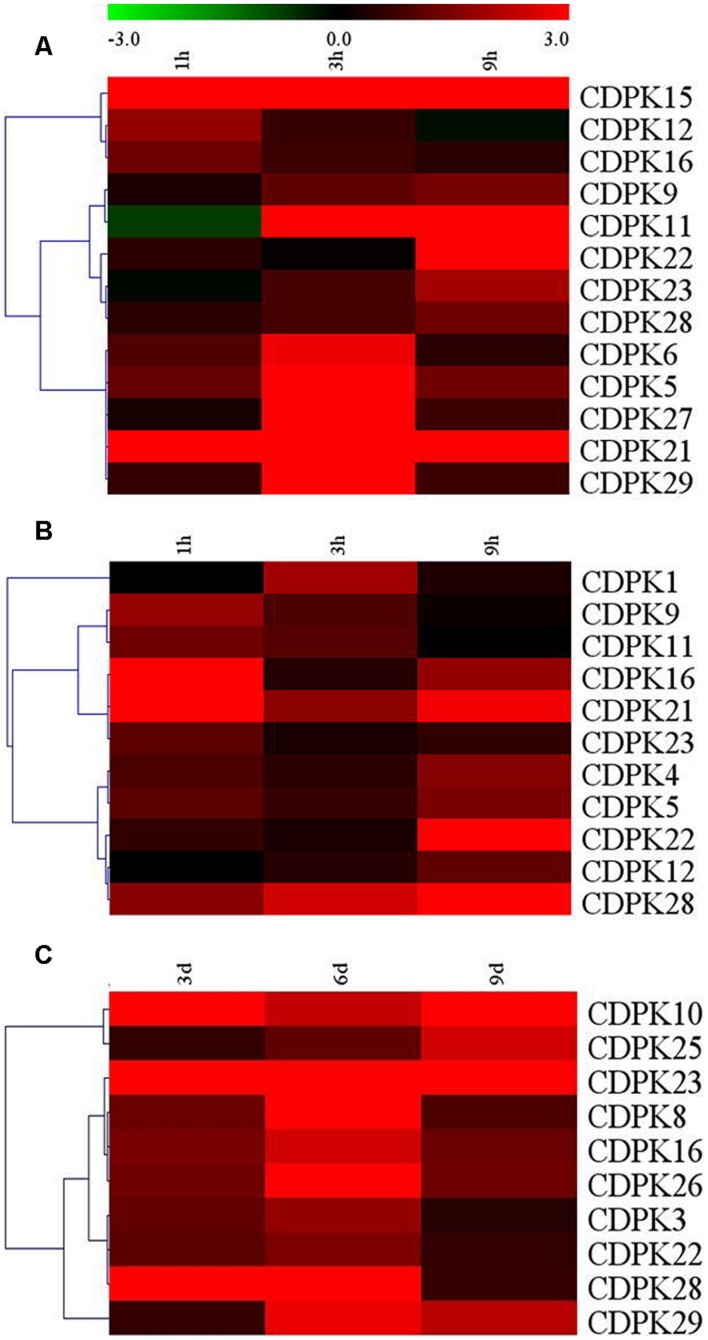
**Up-regulated expression of selected CDPK genes under different abiotic stress conditions. (A)** Upregulated CDPK genes under 45°C; **(B)** Upregulated CDPK genes under 4°C; **(C)** Upregulated CDPK genes under drought. The color scale stands for the relative signal intensity values. Hierarchical clustering was deepened on the data analysis.

The role of CDPKs, plant-specific calcium sensor protein kinases, is well known for their involvement in different aspects of plant response to abiotic stresses. Here, transcripts of these *Sl*CDPK genes were analyzed after exposure to heat, cold, and drought stresses, respectively (**Figure 7**). We found that 13, 11, and 11 *Sl*CDPK genes were induced at the transcriptional level after exposure to heat, cold, and drought stresses, respectively. Significantly, the transcript response of these genes to drought stress was largely similar to their response to ABA treatment, which highly implied the involvement of CDPK-mediated ABA signal pathways in drought response in tomato (Supplementary Figure S1). Among them, *SlCDPK21* was the gene with most highly induced by heat and cold treatments while *SlCDPK23* was the gene with most highly induced by drought treatment, respectively. Other paralogous genes such as *SlCDPK5/*6, *SlCDPK22/27* were highly induced under high temperature. Interestingly, *SlCDPK5/6*, presented similar expression pattern under high temperature stress and drought stress, but quite different pattern at low temperature. These results implied that *SlCDPK* paralogous genes could play a differential role under certain conditions. Thitherto, function of several orthologous CDPK genes in other plant species has been studied. For instance, *AtCPK1*, orthologous to *SlCDPK4/12*, could modulate cold tolerance by altering the phosphoproteome in *Arabidopsis* (Bohmer and Romeis, 2007). Overexpression *OsCPK7* in rice and *AtCPK6* in *Arabidopsis*, respectively, which were homologous with drought-induced *SlCDPK3* from Group I, resulted in enhanced drought tolerance (Saijo et al., 2000; Xu et al., 2010). There are also evidences that *OsCPK9* and *AtCPK10*, which were clustered as an orthologous of highly drought induced *SlCDPK23*, participated in ABA-responsive drought tolerance (Zou et al., 2010; Wei et al., 2014). Function elucidation of these CDPK genes in plant growth, development and stress response in tomato is highly desirable in the future.

## Conclusion

Based on the tomato genome database, a total 29 SlCDPK genes were identified and categorized into four groups. Each of tomato 12 chromosomes was located with more than one *Sl*CDPK variants, which manifested the conservative of *Sl*CDPK family in long evolution. All *Sl*CDPK genes shared the common protein motifs and the exon-intron structures of each *Sl*CDPK genes group showed highly similarity. In addition to the differential transcript levels in different organs, these *Sl*CDPK genes showed quite different response to phytohormones and environmental stimuli. Like drought, ABA upregulated a dozen of *Sl*CDPK genes in transcript level whilst BRs seemed to have only modest effects on the transcript of these CDPKs. In comparison, SA and JA had similar effects on the transcript of these CDPKs. All these results suggested their diverse roles in growth, development, and stress response. The results presented here would be helpful for the better understanding of the evolutionary relationship of this gene family and their biological functions in plant growth, development, and stress response in tomato species.

## Author Contributions

JY and YZ conceived and designed the experiment. ZH and XL performed the experiments. ZH, XX, JZ, and KS carried out the analysis. JY and YZ helped to revise the manuscript. All authors read and approved the final manuscript.

## Conflict of Interest Statement

The authors declare that the research was conducted in the absence of any commercial or financial relationships that could be construed as a potential conflict of interest.

## Footnotes

1 http://www.arabidopsis.org/

2 http://rice.plantbiology.msu.edu/

3 http://www.solgenomics.net

4 http://mips.helmholtz-muenchen.de/plant/tomato/index.jsp

5 http://prosite.expasy.org/scanprosite/

6 http://www.ncbi.nlm.nih.gov/Structure/cdd/wrpsb.cgi

7 http://pfam.sanger.ac.uk/search

8 http://gsds.cbi.pku.edu.cn/
