# Supplementary material for: Genome-Wide Identification and Expression Analysis of Calcium-dependent Protein Kinase in Tomato
Source: Front Plant Sci. 2016 Apr 8;7:469. doi: 10.3389/fpls.2016.00469 (PMC4824780; doi:10.3389/fpls.2016.00469)
Supplement: Supplementary file 1 [file Table_1.DOCX]

**TABLE S1. Gene-specific primers designed for qRT-PCR.**

| Gene name | q-real time PCR forward primer | q-real time PCR reverse primer |
| --- | --- | --- |
| *SlCDPK1* | GACAAGGTCAATTCGGGACT | CTGCCAAATGGTGCATAATC |
| *SlCDPK2* | TGCAGTTCATGTTGTGATGG | AAGCTCAGCAGCCTGTCTCT |
| *SlCDPK3* | CCGAATTGTCTTCCGGTATC | ACCCTTGATGGAAACGATGT |
| *SlCDPK4* | TCTGTTTCAGCTGCAATGTG | TCAGGGTCTTTCCCAGATTC |
| *SlCDPK5* | TGCTTGACAGGAATCCAAAG | CTTTGGATTCCTGTCAAGCA |
| *SlCDPK6* | TCAGATACTGCGAGGCAAAC | TGGGCAGTTAACCTCCTCTT |
| *SlCDPK7* | AGGATCCTAAAGCGCGAATA | ATTCAACACTGCAGATCCCA |
| *SlCDPK8* | TGGCCGTTGATATCTGAGAG | TACTGCAGGATCCAGTGCTC |
| *SlCDPK9* | AAGTCCTGCGAAAGCGTTAT | CTTGCTCAGTTTCATCCCAA |
| *SlCDPK10* | TTCAACTCTGAAGGCGATTG | GGCTTCCAACAACATCATTG |
| *SlCDPK11* | GCGGGTATATTACCGCAGAT | TCCGTCCATCGTTATCTTGA |
| *SlCDPK12* | AGCTGATGTCTGGAGTGCTG | AAGTCAAGATCACCGTGCAG |
| *SlCDPK13* | CTCACCAGGGATCCTAGAGC | GACAGAACTGCAGAACCCAA |
| *SlCDPK14* | TGGAGAAGCACCAGACAC | AACATCCTGCAATAACCC |
| *SlCDPK15* | ACGGACAATAGTGGGACA | TGCTTAACTTCAGCCTCC |
| *SlCDPK16* | TGCTGGAGGAGAGTTGTTTG | TTCTCTGGCTTGAGATCCCT |
| *SlCDPK17* | CTTAGACACGCGATGGAAGA | CGCGACAAACTCATCGTAGT |
| *SlCDPK18* | AGAAGTGGAGCCAAACAACC | CAGAGCTCATCCTGCACATT |
| *SlCDPK19* | AGAGTCGGAAGTCAGGCAGT | TGATCTTCTCTTTCCATGCG |
| *SlCDPK21* | GGAAACAATGACATGCAAGG | TGGCTTTGAAGAATGGTTTG |
| *SlCDPK22* | TACAGACAAGGATGGTCGGA | ATGAGCCTTCCCTCATCAAC |
| *SlCDPK23* | CATGGCCTCAAGTCTCTGAA | TCTCCCAGTGGAACATTTGA |
| *SlCDPK24* | ATGGGACAAACTGTTGCTGA | TTGTAAGTGCACAGCCATTG |
| *SlCDPK25* | CTGCTGTTACACGGACGATT | TTCTCGGGCTTCAAATCTCT |
| *SlCDPK26* | TGATTGCTGAGCATTTGACA | GGAATTTGATGGCCAAGTTT |
| *SlCDPK27* | GCAGCTGCGGTTATTATGAA | GCCTTCAAAGGAGCAGTTTC |
| *SlCDPK28* | AGTTGTTGGGTCATGGACAA | CTGCAATTGGAACAACCATC |
| *SlCDPK29* | TGGGTGGAGAGTTAGGAAGG | AGGCGCTTGTCGATAGACTT |
