# Supplementary material for: Genome-Wide Identification and Expression Analysis of Calcium-dependent Protein Kinase in Tomato
Source: Front Plant Sci. 2016 Apr 8;7:469. doi: 10.3389/fpls.2016.00469 (PMC4824780; doi:10.3389/fpls.2016.00469)

FIGURE S1. Expression analysis of CDPK genes in tomato leaves against different abiotic stress. (A) CDPK genes expression under 45 ºC; **(B)** CDPK genes expression under 4 ºC; **(C)** CDPK genes expression under drought condition. The color scale stands for the relative signal intensity values. Hierarchical clustering was deepened on the data analysis.

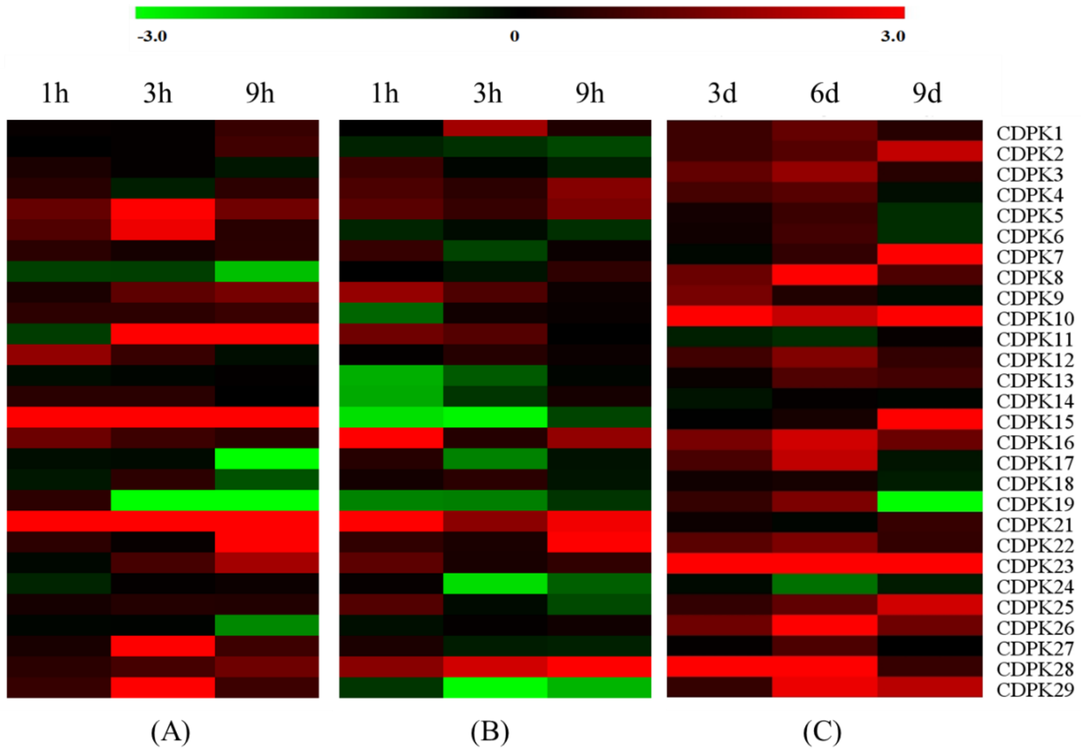

Supplement: Supplementary file 2 [file Image_1.PDF]
